# The interaction of feature and space based orienting within the attention set

**DOI:** 10.3389/fnint.2014.00009

**Published:** 2014-01-30

**Authors:** Ahnate Lim, Scott Sinnett

**Affiliations:** Department of Psychology, University of Hawaii at ManoaHonolulu, HI, USA

**Keywords:** attention, attention set, exogenous, cuing, object feature

## Abstract

The processing of sensory information relies on interacting mechanisms of sustained attention and attentional capture, both of which operate in space and on object features. While evidence indicates that exogenous attentional capture, a mechanism previously understood to be automatic, can be eliminated while concurrently performing a demanding task, we reframe this phenomenon within the theoretical framework of the “attention set” (Most et al., 2005). Consequently, the specific prediction that cuing effects should reappear when feature dimensions of the cue overlap with those in the attention set (i.e., elements of the demanding task) was empirically tested and confirmed using a dual-task paradigm involving both sustained attention and attentional capture, adapted from Santangelo et al. (2007). Participants were required to either detect a centrally presented target presented in a stream of distractors (the primary task), or respond to a spatially cued target (the secondary task). Importantly, the spatial cue could either share features with the target in the centrally presented primary task, or not share any features. Overall, the findings supported the attention set hypothesis showing that a spatial cuing effect was only observed when the peripheral cue shared a feature with objects that were already in the attention set (i.e., the primary task). However, this finding was accompanied by differential attentional orienting dependent on the different types of objects within the attention set, with feature-based orienting occurring for target-related objects, and additional spatial-based orienting for distractor-related objects.

Attention—considered to be a broad and multi-faceted information processing mechanism—contains many seemingly dichotomous yet complementary characteristics, one of which is how it must have the capacity to be both focused and distractible at the same time. The ability to ignore irrelevant stimuli and closely attend to a specific task at hand is fundamental to task-oriented and goal-directed behavior. Conversely, the ability to be distracted by potentially dangerous events, or to be drawn towards relevant information outside the current task or area of focus, is paramount for avoiding harm and responding in a timely manner to the environment. In fact, neurological evidence has demonstrated that these somewhat dissociable top-down and bottom-up mechanisms are underpinned by distinct and interactive neural networks and synchrony frequencies (Sarter et al., 2001; Corbetta and Shulman, 2002; Buschman and Miller, 2007; Corbetta et al., 2008).

On the one hand, many daily tasks can be categorized as goal-directed and involving characteristically top-down control of behavior. Although the environment may contain objects that actively compete for attention, what ultimately becomes selected for processing can be influenced to a large extent by “top-down signals” that filter for behaviorally relevant objects (Desimone and Duncan, 1995). Top-down attention is used in visual search (Wolfe, 2007), endogenous (participant directed) orienting of attention to spatial locations (Posner, 1980), and even feature integration (Treisman and Gelade, 1980), to name a few (but see Theeuwes, 2013 for a bottom-up perspective). On the other hand, the attentional system must also respond effectively to important stimuli (such as impending threats). It has been shown that this more reflexive aspect of attention is often dependent on the particular nature of environmental circumstances at hand. Such factors may include for example, the role of stimulus saliency (Jonides and Yantis, 1988), the role of spatial location (Posner et al., 1980; LaBerge, 1981; Cave and Pashler, 1995; Lu and Dosher, 2000; Mathôt and Theeuwes, 2010a,b), as well as the object features. Other factors, such as object relevancy to behavioral goals (e.g., Yantis and Egeth, 1999), may be less clear cut as some researchers classify them as bottom-up and others as top-down. Furthermore, the argument can often be made that top-down explanations—such as feature-based attending, for example—could in fact be supplanted by bottom-up *priming* explanations (where in this case attending to a particular feature causes enhanced processing for that feature across the whole visual field, see Awh et al., 2012).

## Automaticity of attentional orienting

One extensively studied aspect of attention that is historically viewed as a bottom-up process is the spatial capture of attention (ala Posner cuing, see Posner, 1980). More recent evidence has shown however, that even attentional capture can at times be interrupted when an observer is undergoing a difficult and demanding task. Specifically, where such exogenous, or stimulus-driven, mechanisms were previously thought to be automatic (Müller and Rabbitt, 1989), attentional orienting may in fact be eliminated in a state of focused attention. For example, several studies have demonstrated that requiring participants to perform a concurrent demanding task can effectively eliminate the ability of exogenous cues to capture attention (Yantis and Jonides, 1990; Theeuwes, 1991; Santangelo et al., 2007; Santangelo and Spence, 2008; Santangelo et al., 2008). Two of these studies, for instance, employed central-arrows as 100% predictive cues in a target detection task, while also deploying abrupt visual onsets as exogenous cues (Yantis and Jonides, 1990; Theeuwes, 1991), and found that the abrupt visual onsets had no effect on performance. Yet a different study by Van der Lubbe and Postma (2005) used more eccentric (peripheral) exogenous cues and obtained evidence to the contrary, where effects of the abrupt visual onsets were observed even when attention was engaged. Of direct interest here, the elimination of the exogenous cuing effect has only been observed when presenting cues and targets in the periphery, while directing attention to a central task. Regardless, there appears to be evidence indicating that under some circumstances exogenous cuing effects remain, while under others these effects can be eliminated. However, the exact roles and importance of the relationship between objects in the periphery and objects in the central task have yet to be explored. In examining these issues, and reconciling such dichotomies as the top-down and bottom-up views, it may be helpful to adopt a theoretical framework that can predict what dimension or mechanism will be most relevant at any given point in time, and under what type of circumstances.

## The attention set framework

Although it is inherently difficult to formulate theories of attention that are both broad in scope (encompassing several classes of phenomena) while concurrently possessing predictive power for detailed behavioral outcomes, there are frameworks that could provide initial scaffolding towards such comprehensive theories. One such general framework for combining aspects of both inattentional blindness (i.e., an indirect measure of sustained focus) and attentional capture has been proposed by Most et al. (2005). Central to their theoretical framework, is the idea of an “attention set” that is synonymous with the current task at hand or state of mind. The authors postulate that this “attention set” should be the most influential factor in determining what captures attention. In broad strokes, this attention set could include a category of objects that direct or attract attention as needed, potentially overriding any spatial-based or more bottom-up aspects of attention. Incidentally, the idea that the current frame of mind determines how attention is allocated has been around for some time, with one of the first instantiations being Neisser’s (1976) construct of a *perceptual cycle*. While Most and colleagues’ formulation provides an explanatory construct for both sustained attention and attentional capture, their emphasis on the attention set can be used to infer predictions. Specifically, Most et al. (2005, p. 218) claim that:

“Although some stimulus properties (e.g., uniqueness) can affect noticing, to a larger extent the unexpected objects that people consciously see depend on the ways in which they “tune” their attention for processing of specific types of stimuli—that is, on the attentional set that they adopt.”

Consequently, this leads to the prediction that objects that are irrelevant, but are nevertheless within the same attention set (i.e., the objects share some specific category or feature), should be capable of capturing attention (i.e., irrelevant objects that are separate but similar to the targets used in an attended to task will capture attention), whereas events that fall outside of the attention set should go unnoticed (e.g., a gorilla walking amidst a group of people passing a basketball while counting passes, see for example Simons and Chabris, 1999). This latter prediction could be used to explain the results of Santangelo et al. (2007), who demonstrated that the exogenous cuing effect is eliminated when simultaneously participating in a difficult central task. It is important to note that the cues used in this task fell outside of the attention set of the central task.

Most et al.’s (2001, 2005) predictions regarding the influence of the attention set were supported by a series of empirical studies centered around a paradigm in which participants counted the number of bounces of a subset of items moving within a display. Crucially, an unexpected object entered the display after several trials and detection rates for these objects were used as a measure of attentional capture. In this way, the experimenters were able to manipulate the composition of the attention set (the objects moving and bouncing within the display), and observe subsequent effects on attentional capture. Of critical importance to their theory, the findings suggested that the capture of awareness is influenced both by top-down and bottom-up interactions, where the most influential factor is ultimately the attention set adopted (although certain bottom-up factors such as stimulus salience can increase the chance that objects will be noticed). In general, when unexpected objects possessed features that overlapped with those in the attentional set, participants consistently noticed them (despite the separation in space), whereas when the items were outside the attention set, participants rarely noticed them. Bearing in mind that Most et al.’s (2001, 2005) experiments were adaptations of an inattentional blindness paradigm where participants were tested on their awareness and processing of an unexpected event, the question remains as to whether the same predictions would generalize to a different task setting where attention is focused on a central area (rather than across the experimental display), and attentional capture is systematically measured through exogenous cuing rather than self-reported conscious detection.

To recall an earlier mentioned related example in more detail, Santangelo et al. (2007) devised a paradigm involving both a demanding central task and an exogenously cued target detection task, and found that exogenous orienting does not capture attention in a mandatory fashion when undergoing a demanding central task (see Santangelo and Spence, 2007, 2008; Santangelo et al., 2007). That is, when one’s attention is engaged in performing a demanding task, the automatic effects of exogenous cues seemingly disappear. This finding is especially important considering that previous accounts of exogenous cuing suggest that peripheral cues automatically capture attention (Jonides, 1981; Müller and Rabbitt, 1989; Van der Lubbe and Postma, 2005).

While it is possible that the elimination of the cuing effect could be related to an increase in perceptual load and a concomitant reduction in available attentional resources (as suggested by Santangelo et al., 2007), Most et al.’s (2005) theoretical framework could equally predict the same result. That is, Most et al. would predict that elimination of the cuing effect would be related to the fact that the peripheral cues were objects that were not contained in the “attention set” (i.e., the cue was not a part of, nor was it related to, anything in the central task). This was precisely the case in the paradigm used by Santangelo et al. (2007, 2008). Specifically, participants were required to detect a number amongst a rapid serial presentation of letters and numbers, while the peripheral cue was a geometric shape (i.e., not a number). Adopting Most et al.’s logic, the peripheral cue was task irrelevant and not related to anything in the attention set (letters or numbers), therefore it is not surprising that it failed to capture attention. Accordingly, one could predict that if the irrelevant peripheral cues were to be manipulated such that they overlapped with objects in the current attention set (i.e., the peripheral cues and central targets contain objects that come from the same category or share the same features), they should successfully capture attention despite being completely irrelevant to the task at hand. While such a result would normally be construed as evidence for a space based attentional mechanism (i.e., typical cuing effect for the cued side), it is important to note that the crucial manipulation of aligning features of targets with cues in order to obtain such a cuing effect would thereby imply that feature-based attention is responsible for such an effect. Given that attention can be directed to both features and space, understanding how the attention set can predict peripheral attentional capture requires understanding how feature-and spatial-based attention operates as well.

## Feature and space-based attention

Both spatial and featural information are fundamental to our interaction with the environment, with studies showing that attention can be aligned along both dimensions (Maljkovic and Nakayama, 1994, 1996). Furthermore, their interaction is also important as demonstrated in a study by Baylis and Driver (1992), where it was found that the influence of target processing on distractor processing was affected not only by the featural overlap of the items, but also by spatial factors such as proximity and Gestalt principles of perceptual organization. Although the relationship between feature-based and spatial-based attention is a topic that has gained more interest recently, historically speaking space based theories of attention dominated up until the early 1980s (Eriksen and Hoffman, 1972; Eriksen and Eriksen, 1974; Posner et al., 1980; Hoffman and Nelson, 1981; LaBerge, 1983; Yantis and Johnston, 1990). After this point, increasingly more research began to point towards an attentional mechanism attuned to objects and their properties, rather than solely to space (Prinzmetal, 1981; Duncan, 1984; Egly et al., 1994; Egeth and Yantis, 1997). Although Posner’s widely influential research (Posner et al., 1980; Posner and Cohen, 1984) suggested a special role for spatial information in attention (in comparison to which other non-spatial features are afforded a lesser role), many studies since then have demonstrated the importance of featural information. Indeed, attentional selection according to features is important in many theories of attention (Duncan and Humphreys, 1989; Bundesen, 1990; Serences and Boynton, 2007; Wolfe, 2007). Overall, as more evidence has accrued since the 1980s, feature-based processing is now understood to be a fundamental aspect of attention, as it operates in parallel across the entire visual field (Treue and Martinez-Trujillo, 1999; Saenz et al., 2002; Bichot et al., 2005), is independent from spatial attention (Zhang and Luck, 2009), may actually prioritize task relevant features over other factors such as sensory similarity (Bondarenko et al., 2012), and may even have neural onsets that occur as early as spatial processing (Zhang and Luck, 2009).

Although both feature and spatial-based attention are important, the fact that the vast majority of experimental paradigms are necessarily grounded in both properties can make the task of determining when each dimension is most relevant rather complicated. Using the attention set theory (Most et al., 2005) in the context of the previously described dual task paradigm (see Santangelo et al., 2007) enables one to investigate the processing of central objects and its relationship to attentional capture (exogenous orienting) for peripheral objects (and how this relationship is mediated by the attention set). Additionally, this approach also allows us to investigate the interaction of feature and spatial-based attention. Using a within subjects design, participants performed a difficult central task requiring them to detect numbers that were presented within a stream of rapidly presented letters. On a subset of trials, participants responded to the location (above or below) of a peripherally presented target that was orthogonally cued (right or left). Critically, we presented different types of peripheral cues in Experiment 1, such that the cue either shared features with the target objects in the central task, or did not have any commonalities with those objects. Note that the cue itself was completely irrelevant to the task and in theory would be outside of the attention set (i.e., the central task in this case). If our interpretation of Most et al.’s (2005) prediction holds, exogenous cuing effects should be eliminated when peripheral cues do not share any common features with objects in the central task. However, if peripheral cues are related to (or were even subsets of) the objects in the central task, then an exogenous cuing effect (i.e., attentional capture) should emerge.

## Experiment 1

### Method

#### Participants

Thirty participants were recruited from undergraduate courses at the University of Hawaii at Manoa. Five participants were excluded from the analysis,^1^ resulting in a final sample of 25 participants (mean age = 22 ± 4; 15 females) for the analysis. All participants were naïve as to the purpose of the experiment and had normal or corrected to normal vision. Participants were offered course credit for their participation, and informed consent was obtained from all. Ethical approval was obtained from the University’s Committee on Human Subjects.

#### Stimuli

**Figure 1:**
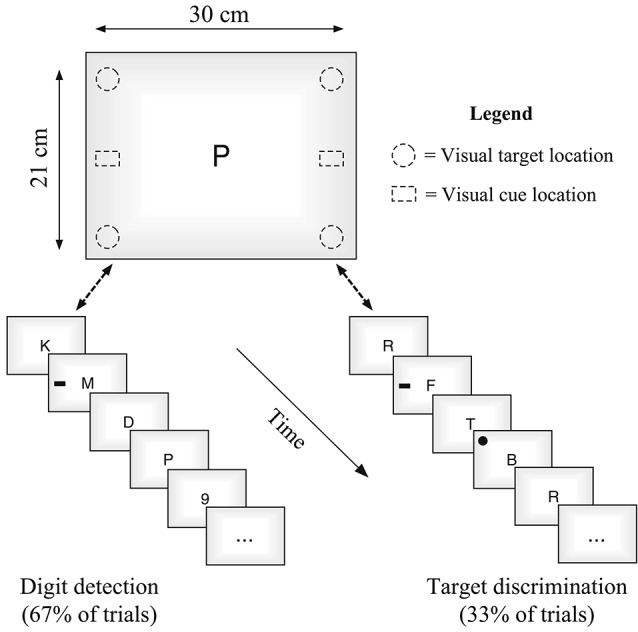
**Schematic representation of the task.** Both types of trials (digit detection occurring 2/3 of the time, and spatial detection occurring 1/3 of the time) were interleaved and randomized, with cues being presented on every trial, in either the third or sixth position in the stream.

**Figure 2:**
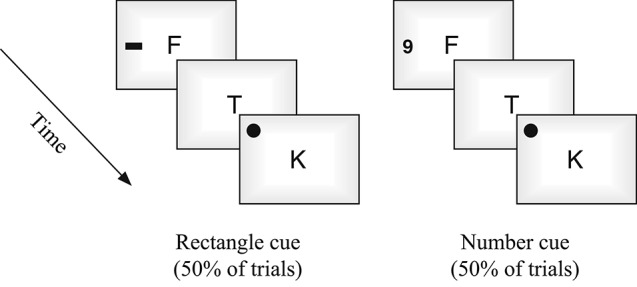
**The two different cue types used in the task in Experiment 1**.

All stimuli were presented on a 20′, iMac using Bootcamp and DMDX software (Forster and Forster, 2003). Observers sat approximately 60 cm from the display. Stimuli in the central rapid serial visual presentation (RSVP) stream was constructed from randomly chosen non-repeated letters (11 selected from set of 17: B, C, D, E, F, J, K, L, M, N, P, R, S, T, Y, X, Z), each presented for 100 ms with a 16.7 ms inter-stimulus interval (ISI). For digit detection trials, numbers were selected from a set of six: 2, 3, 4, 5, 6, 9. Visual targets were black circles (subtending 2°) and cues were either black rectangles (2.5° × 1.7°) or numbers of comparable dimensions (i.e., outside or in the attention set of the primary task, respectively; see Figures 1, 2). Aside from the use of number cues on half of the trials, all stimuli, presentation times, and counterbalancing were constructed to be similar to the unimodal visual condition used in Santangelo et al.’s experiment (2007).

#### Procedure

All participants were presented with written instructions for the task on the computer screen. Next, they were presented with practice trials and given accuracy and reaction time (RT) feedback after the end of each trial. Participants had the option of repeating the instructions as well as repeating the practice trials before they started the actual experiment. The experimenter also monitored participants and their performance during the practice trials to ensure proper understanding of the task.

Participants were required to monitor the RSVP stream presented in the center of the display, and to respond to the occurrence of a numerical digit. A digit occurred on the majority of trials (67%). On the remaining trials (33%) the digit was not presented and instead, participants responded to the location of a spatial target that could have occurred in one of the four corners of the screen. A peripheral cue was presented on all trials, but was irrelevant to either task. The cue could have validly predicted the side of the spatial target or not (note, a spatial target was not present on digit trials). Responses were made using one of three keys following detection of either: (1) a number; (2) an upward spatial target; or (3) a downward spatial target.

Each trial began with a fixation cross (1000 ms) followed by the RSVP stream of 11 items. On digit detection trials, the numbers randomly occurred in either the third, sixth, or ninth position in the stream (see also, Santangelo et al., 2007). A spatial cue was also presented on each trial (for 100 ms, identical to item duration), occurring in the third or sixth position on either the right or left side of the display equiprobably. When spatial targets occurred a number was not presented in the stream, and the spatial target appeared two positions after the cue (5th or 8th position). The two types of cues, rectangles or numbers, also occurred equiprobably (see Figure 2). Each experimental session consisted of 196 randomized trials, 132 of which were the digit detection task, and 64 of which were target detection (Santangelo et al., 2007). Cue combinations and trial repetitions were counterbalanced. Participants were instructed to respond as soon as targets were detected.

### Results

Mean RTs and accuracy rates were analyzed using three repeated measures ANOVAs (analysis of variance): one for the overall experiment and two separate ANOVAs for the digit and spatial target detection conditions. Assumptions of sphericity were tested on all analyses, with Huyn-Feldt corrections being applied to *p* values where appropriate.

**Figure 3:**
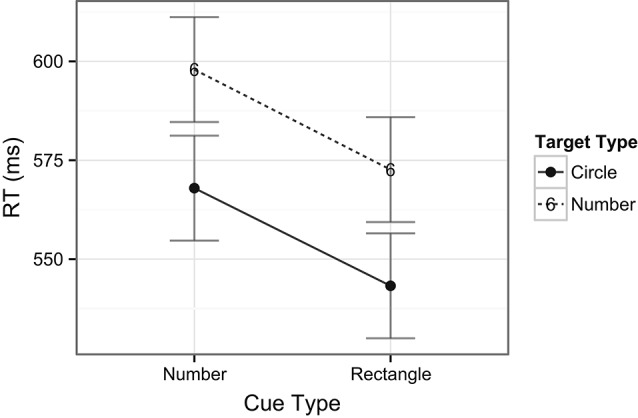
**Mean RTs across tasks and cue types.** Note that number cues shared object properties with the number detection task, whereas rectangle cues did not share object properties with the task. Error bars indicate Fisher’s Least Significant Difference (FLSD) for the plotted effect.

**Figure 4:**
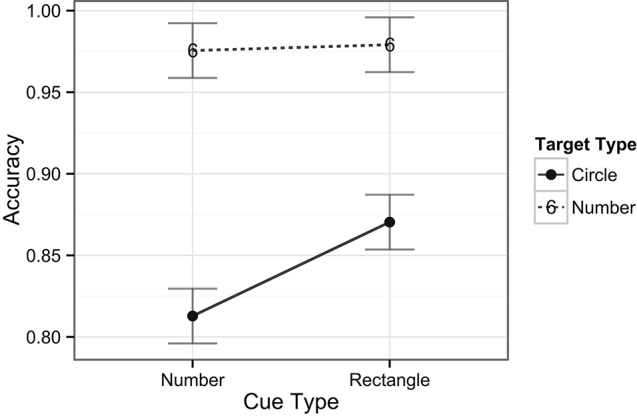
**Mean accuracy rates across tasks and cue types.** Plotted bars indicate Fisher’s Least Significant Difference (FLSD, similarly for all subsequent graphs).

The ANOVA performed on the RT data included the factors of task type (digit or target) and cue type (rectangle or number). There was no main effect of task type, *F*(1, 24) < 1, *ns*, indicating that there were no overall differences in RTs across digit (i.e., central; *M* = 585 ms) and target (i.e., peripheral; *M* = 556 ms) detection tasks. There was, however, a main effect of cue type, *F*(1, 24) = 8.8, *p* = 0.007, indicating that, overall, RTs were slower when number cues (*M* = 583 ms) occurred compared to rectangle cues (*M* = 558 ms) in the exogenous cuing task. There was no interaction between task and cue types, *F*(1, 22) < 1, *ns*, indicating no differences in RT patterns across the two tasks (see Figure 3). In examining the accuracy data, there was a main effect of task type, *F*(1, 24) = 44.8, *p* < 0.001, with higher accuracy for the digit (central) task (98%) compared to the target (peripheral) task (85%). A main effect of cue type revealed that accuracy was lower on trials with number cues (89%) than on those with rectangle cues (92%), *F*(1, 24) = 6.4, *p* = 0.02, indicating that on average the task was more difficult when number cues were present, in line with the previously mentioned longer response latencies for this comparison. Notably, the analysis also revealed a significant interaction between task and cue types, *F*(1, 24) = 5.5, *p* = 0.03, indicating that number cues tended to be more distracting than rectangle cues (81% vs. 87%, respectively) during spatial target detection, but not during digit detection (98% for both type of cues, see Figure 4).

**Figure 5:**
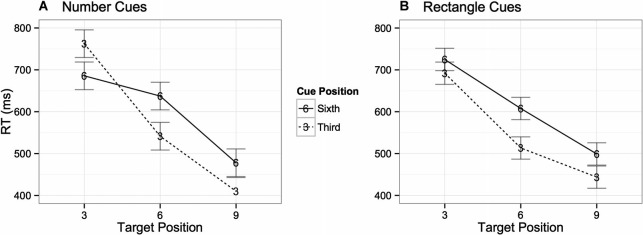
**Interaction between target position, cue position in temporal stream, and cue type.** Graph **(A)** shows trials with number cues, whereas Graph **(B)** shows those with letter cues.

A three way ANOVA with factors of digit (target) position (3), cue position (2), and cue type (2) performed on the digit detection condition revealed that participants detected the digits significantly faster when they were presented in the ninth (*M* = 458 ms) position in the central task than when presented in the sixth (*M* = 570 ms) or third (*M* = 710 ms) positions respectively, *F*(2, 48) = 23.8, *p* < 0.001. RTs were also faster when cues were presented in the third position (*M* = 564 ms) than in the sixth position (*M* = 603 ms), *F*(1, 24) = 12.7, *p* = 0.002. There was also a significant interaction between digit position and cue position, *F*(2, 48) = 6.2, *p* = 0.004, suggesting that performance was poorer when the cue occurred at the same time as the digit. Although there was no main effect in mean RTs between trials with number cues compared to trials with rectangle cues *F*(1, 24) < 1, *ns*, there was a significant three-way interaction, *F*(2, 48) = 3.7, *p* = 0.04, indicating a different pattern of RTs between rectangle and number cues. Specifically, when the target and cue both occur in the third position the number cues adversely affected performance whereas the rectangle cues did not (see Figure 5). No significant differences were found when examining accuracy rates across digit positions.

**Figure 6:**
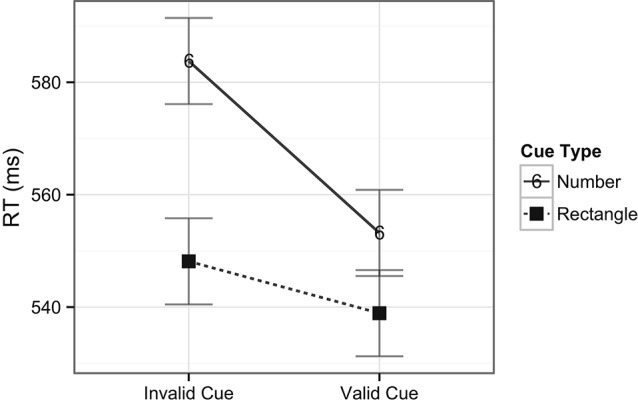
**Interaction of cuing effects within the spatial target detection condition**.

Lastly, a two way ANOVA was performed on the spatial target detection condition with factors of cue validity (2) and cue type (2). While the main effects of cue validity on RTs were only marginally significant with a trend towards faster RTs for valid cues compared to invalid cues, *F*(1, 24) = 3.4, *p* = 0.08, there was a stronger main effect of cue type, *F*(1, 24) = 10.2, *p* = 0.004, where RTs were slower when the target preceding cues were numbers (*M* = 568 ms) compared to when they were rectangles (*M* = 544 ms). Importantly, the interaction between cue validity and cue type was also significant, *F*(1, 24) = 4.1, *p* = 0.05, indicating the presence of cuing effects for number cues on the one hand (553 ms for valid cues, and 584 ms for invalid cues), and the lack of cuing effects for rectangle cues on the other hand (539 ms for valid cues, and 548 ms for invalid cues, see Figure 6).

**Figure 7:**
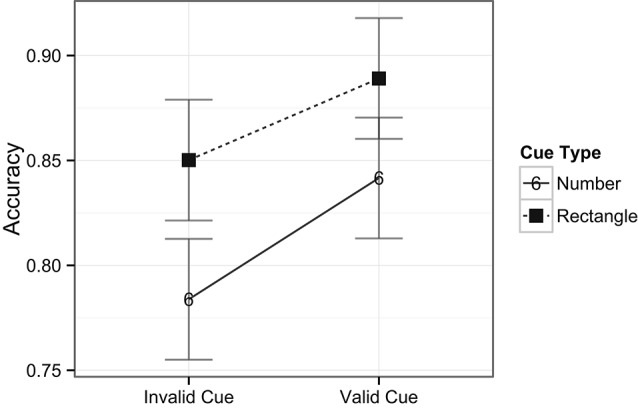
**Accuracy across the spatial target detection condition**.

For the accuracy data, there was a main effect of cue validity, *F*(1, 24) = 5.6, *p* = 0.03, with higher accuracy for valid cues (87%) compared to invalid cues (82%). Accuracy was lower on trials with number cues (81%) than on those with rectangle cues (87%), *F*(1, 24) = 5.9, *p* = 0.02, indicating that performing the spatial task was more difficult when number cues were present than with rectangle cues. The interaction between cue validity and cue types was not significant, *F*(1, 24) < 1, *ns* (See Figure 7).

### Discussion

The main purpose of Experiment 1 was to test Most et al.’s (2005) theoretical framework on attention by examining the effects of target and cue object properties on the peripheral capture of attention. To this end, our findings In Experiment 1 indicate that irrelevant items that fall within the attention set are capable of capturing attention while irrelevant items that fall outside of the attention set do not. That is, as was predicted, peripheral cues that were in the same category, or had overlapping features with the central task (numbers) had a cuing effect (31 ms) on spatial target detection, while items outside the category (rectangles) had a non-significant cuing effect (9 ms; i.e., the cuing effect was eliminated). Not only was a cuing effect observed for peripheral number distractors, but this type of cue also led to a general increase in RTs for spatial trials. This indicates that despite being irrelevant to the task, the number cues were processed and served as more effective distractors than the rectangles. In a sense, sharing object properties between cue and target (i.e., both fell within the attention set) seemed to drive a spatial cuing effect, which was eliminated when the cues did not share object properties with the target (i.e., outside of the attention set).

Although valid number cues effectively captured spatial attention, they did not facilitate overall faster RTs. That is, the mean RT for trials with rectangle cues was in fact faster than for number cues, despite the lack of a cuing effect in this condition. This may possibly be due to the interaction of the number cues with the requirements of the central task. That is, any potential facilitating effects on performance of the valid number cues were probably offset by the overlap between objects in the central and peripheral tasks, possibly leading to interference. Further evidence for this interference was also observed in the higher error rates for trials with number cues when compared to rectangle cues for spatial target detection (Figure 3).

RTs on the digit detection trials also suggest greater interference from the number cues. The interaction indicates that performance was worse on trials when the cue occurred at the same time as the digit, with more interference occurring from number cues when presented in the third frame. It is worth noting here that the lack of clearly distinguished differences between the effects of the rectangle and number cues on digit detection may be due to a more general distracting effect of the number cues. That is, the number cue may induce a distracting effect that generalizes beyond those particular trials to even cause the rectangle cues to become more distracting than they naturally would be.

Aside from providing support for the theoretical position that the most influential factor for attentional capture is the “attention set”—or the current items in focus—our findings also lend support to the notion that feature based attention can interact with space based attention (e.g., Duncan, 1984; Egly et al., 1994; for a review, see Scholl, 2001). That is, the spatial cuing effect was only observed when the cue was in fact an object that shared features with the targets in the attention set. In this way, the spatial cuing effect could arguably be driven by feature-based attention. In refining our understanding of the attention set, it becomes imperative to more precisely define the attention set itself, for the reason that when one is engaged in a task, there are usually multiple objects or different classes of events to attend to. For example, in this experiment, we defined the attention set as being the digit detection task, due to the fact this occurred the majority (67%) of the time. The most important object in the central stream was the number, and accordingly the identity of peripheral cues was manipulated to be numbers on half of the trials.^2^ The question remains however, as to what role the irrelevant objects in the central task (i.e., the letters) play within the attention set.

Despite the fact that the letters within the RSVP stream are of a different category than the number targets, they are nevertheless processed by virtue of proximity to the number targets (both temporally and spatially) and the fact that participants must monitor the stream in order to accurately detect the number amongst letters. Thus it is possible that the letters could also be considered as falling within the attention set, however, due to the nature of the RSVP task the letters might be processed differently than the numbers. For this reason a peripheral cue that is a letter might not capture attention the way number cues did in Experiment 1. The question remains in this case as to the precise role of object category in the attention set, and whether the letters are afforded a similar or different role than the numbers. Thus, the purpose of Experiment 2 was to answer these questions through an analogous experiment that replaced the number cues used in Experiment 1 with letter cues in order to determine any differences in performance patterns.

## Experiment 2

### Method

#### Participants

Thirty naïve participants were recruited in an identical manner to Experiment 1. Informed consent was obtained from all participants. An identical performance exclusion criteria was applied as in Experiment 1, resulting in a sample of 29 subjects (mean age = 21 ± 2; 20 females) for the final analysis.

#### Stimuli/Procedure

**Figure 8:**
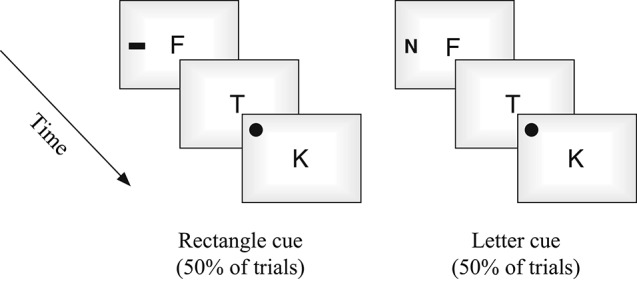
**The two different cue types used in Experiment 2**.

All stimuli were constructed and presented identically as in Experiment 1 with the exception that the number cues used in Experiment 1 (on 50% of the trials) were now replaced with letter cues (see Figure 8). The letter cues were selected from the same subset of 17 letters that the central stream was selected from, with the sole criteria that the cue did not overlap with any of the other letters in the central stream on that trial. The experimental procedure was identical to Experiment 1.

### Results

**Figure 9:**
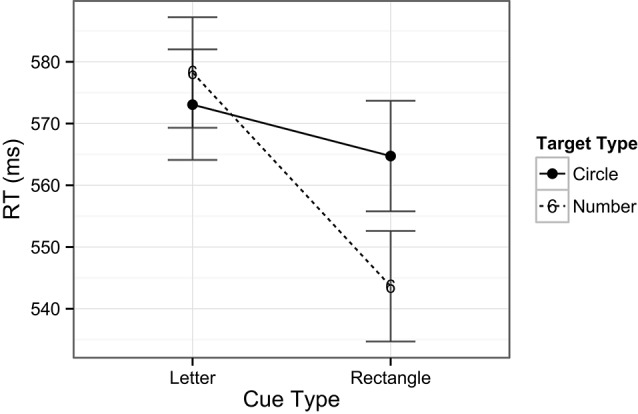
**Mean RTs across tasks and cue types**.

Similar to Experiment 1, mean RTs and error rates in Experiment 2 were analyzed using three repeated measures ANOVAs. RT data was analyzed with a two factor ANOVA with factors of task type (digit or target) and cue type (rectangle or number). There was no main effect of task type, *F*(1, 28) < 1, *ns*, indicating that there were no overall differences in RTs across digit (central; *M* = 585 ms) and target (peripheral; *M* = 556 ms) detection tasks. There was, however, a main effect of cue type, *F*(1, 28) = 15.6, *p* < 0.001, indicating that, overall, RTs were slower when letter cues (*M* = 576 ms) occurred compared to rectangle cues (*M* = 554 ms). There was also a significant interaction between task and cue types, *F*(1, 28) = 4.5, *p* = 0.04, indicating that letter cues tended to be more distracting than rectangle cues (578 ms vs. 544 ms) during digit detection (despite being irrelevant to the task), but not during spatial target detection (573 ms vs. 565 ms, see Figure 9).

**Figure 10:**
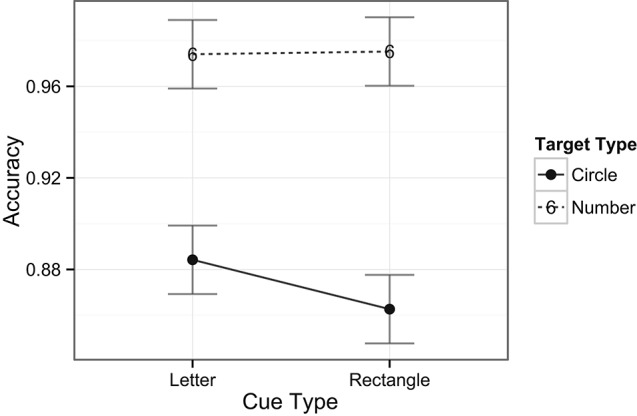
**Mean accuracy rates across tasks and cue types**.

In examining the accuracy data, there was a main effect of task type, *F*(1, 28) = 50.8, *p* < 0.001, with higher accuracy rates for the digit task (97%) compared to the target task (87%). Neither was there a main effect of cue type nor were there any interaction effects between task and cue types for accuracy data (see Figure 10).

**Figure 11:**
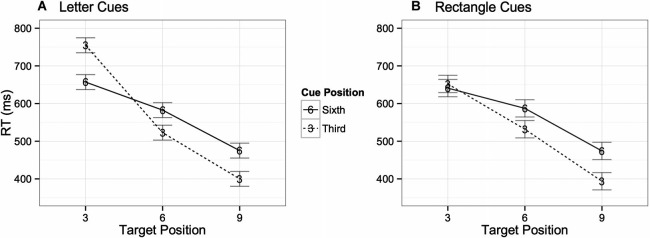
**Interaction between target position, cue position in temporal stream, and cue type.** Graph **(A)** shows trials with letter cues, whereas Graph **(B)** shows those with rectangle cues.

A three way ANOVA with factors of digit position (3), cue position (2), and cue type (2) was performed on the digit detection condition and revealed that participants detected the digits significantly faster when they were presented in the ninth (*M* = 442 ms) position than when presented in the sixth (*M* = 553 ms) or third (*M* = 673 ms) positions respectively, *F*(2, 56) = 48.9, *p* < 0.001. RTs were marginally faster when cues were presented in the third position (*M* = 550 ms) than in the sixth position (*M* = 567 ms), *F*(1, 28) = 3.6, *p* = 0.07. There was also a significant interaction between digit position and cue position, *F*(2, 56) = 18.1, *p* < 0.001, suggesting that performance was worse when the cue occurred at the same time as the digit. There was a main effect on mean RTs between trials with letter cues compared to trials with rectangle cues, *F*(1, 28) = 4.4, *p* = 0.04, as well as a significant three-way interaction between all factors, *F*(2, 56) = 3.5, *p* = 0.04, indicating different patterns of RTs between rectangle and letter cues. Analogous to Experiment 1, when the target and cue both occur in the third position the letter (number in Experiment 1) cue adversely affected performance whereas the rectangle cue did not (see Figure 11). No significant differences were found in the accuracy rate data.

**Figure 12:**
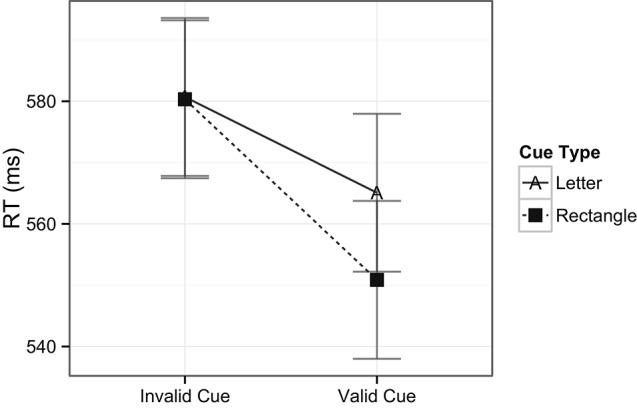
**Cuing effects within the spatial target detection condition**.

A two way ANOVA was performed on the spatial target detection condition with factors of cue validity (2) and cue type (2). Cue validity had a main effect on RTs with faster RTs for valid cues (558 ms) compared to invalid cues (581 ms), *F*(1, 28) = 5.7, *p* = 0.02. Unlike Experiment 1 however, neither was there a main effect of cue type nor were there any interaction effects between cue validity and cue type, both *F*(1, 28) < 1, *ns* (see Figure 12). Also unlike Experiment 1, no significant differences were found in the accuracy rates across cue type or cue validity.

### Discussion

Two major trends can be seen from the results of Experiment 2. First, as predicted, spatial cuing effects were seen on trials with letter cues, similar to the trials with number cues in Experiment 1. The crucial difference observed here—and not in Experiment 1—was the additional cuing effect for rectangles. The main effect of cue validity (for both letter and rectangle cues), and lack of main effect for cue type and interactions indicate similar patterns of performance for trials with either letter or rectangle cues. Interestingly, it would appear that the introduction of letter cues in Experiment 2 somehow lead to a concomitant generalization or mapping to rectangle cues, unlike in Experiment 1 where cuing effects were limited only to the object category of numbers. It is also worth noting here the difference in performance compared to that obtained by Santangelo et al. (2007) using only peripheral rectangle cues. That is, in their study no cuing effects were found, suggesting that merely introducing letter cues on half of the trials can be enough to bring back such cuing effects as observed here.

The second trend observed across task conditions was a slow down in response latency when letter cues were present, especially in the digit detection condition. This suggests that despite being irrelevant to the central task, letter cues nevertheless captured attention. This may be in line with evidence from studies showing that distractors (non-targets) within RSVP streams are indeed processed at a lower level, even if they cannot be consciously recalled (Luck et al., 1996), and that irrelevant peripheral objects can also lead to neural responses associated with executive control mechanisms (Kopp et al., 1996).

## General discussion

One of the main purposes of this study was to test Most et al.’s (2005) prediction that the theoretical construct of the attention set can be an effective conceptual tool in determining what type of features can capture attention when it is directed to separate central and peripheral tasks. To this end, the prediction was partially confirmed as both number and letter peripheral cues (Experiments 1 and 2 respectively, constructed to contain overlapping features with the primary central task) elicited cuing effects. Yet the cuing effect for rectangles (considered to exist outside the attention set) observed only in Experiment 2 raises important questions as to how object properties may influence the specific allocation of attention.

First and foremost, the pattern of results obtained in Experiment 1 aligns with findings related to feature-based attention. That is, the spatial cuing effect appears to be driven solely by whether the cue shares features with the target objects of the primary task. Indeed, the presence of a cuing effect only for number cues that fell within the attention set (and not for rectangle cues in Experiment 1) is in line with findings that the brain regulates attentional control for task-relevant features by amplifying representations of such features, and not by inhibiting task-irrelevant ones (Egner and Hirsch, 2005). The task-relevant nature of numbers may have caused amplification of numerical representations, thereby enabling number cues to capture attention.

**Figure 13:**
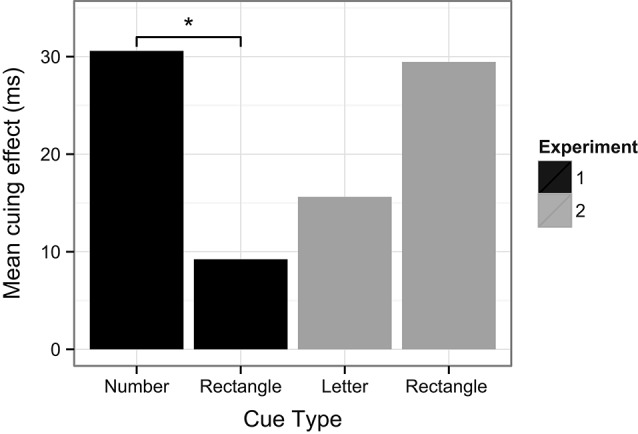
**Cuing effects for the different types of cues used in the target detection trials in Experiment 1 and 2.** Asterisk denotes significance at *p* = 0.05. Note that the cuing effects for letter and rectangle cues in Experiment 2 were not significantly different.

The question remains however, as to what may have caused the rectangle cues to capture attention in Experiment 2 and not in Experiment 1 (see Figure 13). Given that the only actual difference between Experiment 1 and 2 are the object properties of the cues (numbers vs. letters), it logically follows that the properties of letter cues in Experiment 2 are responsible in some way for this effect. The question then, is how did the presence of the letter cues influence the rectangle cues to also capture attention? Importantly, the only commonality between both cues was their spatial location. That is, the cues appeared in identical peripheral locations, at either the right or left side of the display. Thus, it is possible that some mechanism of spatial processing, perhaps the grouping of cues along their common spatial properties, could have been responsible for the pattern of results observed here. Nevertheless, such allocation of spatial attention is speculative, and further experimentation is needed to better support such explanations.

While the results observed in Experiment 1 provides more direct support for the attention set hypothesis, the results from Experiment 2 are less straightforward. Moreover, it should be noted that the generalized capture of attention for both letter and rectangle cues in Experiment 2 may not necessarily be contradictory to the attention set framework. Where the attention set hypothesis predicts that the most important factor in determining attentional capture is how attention is tuned (in this case to the number targets), we do see the strongest cuing effect for numbers (and a corresponding lack of cuing effect for rectangles) in Experiment 1. The presence of the smaller cuing effect in Experiment 2, which was also less differentiated and more generalized (observed for both letters and rectangles cues) could be due to a different mechanism than in Experiment 1.

When considering the role of object features within the attention set, a critical question is whether irrelevant items (in this case, the letters in the central stream, and correspondingly, the letter cues in Experiment 2) are in fact suppressed or inhibited during the dual-task. The cuing effects obtained from both letter and rectangle cues in Experiment 2 indicate that these stimuli were likely not inhibited, but were in fact facilitated. While the results from Experiment 1 are in line with evidence that selective attention operates through cortical signal amplification, recent challenges to the notion of active inhibition—specifically the lack of evidence for inhibition of task-irrelevant items at a neurological level (e.g., Egner and Hirsch, 2005)—are consistent with the seeming lack of inhibition for letters (as well as rectangles) in Experiment 2 (for arguments against inhibition as an attentional mechanism, see Aron, 2007; Hillyard and Anllo-Vento, 1998; Miller and Cohen, 2001; Miller and D‘Esposito, 2005).

It is worth noting how the different rates of occurrence for numbers and letters in the central stream could have influenced how the corresponding cues were processed. That is, within the complete experimental duration for each participant, the central number targets occurred only 132 times, whereas the central letter distractors occurred 2024 times. Given that the letters were displayed much more frequently (15 times more often) than numbers, it is possible this higher frequency may have led to concomitantly higher rates of processing (e.g., neuronal firing) in peripheral spatial locations which could have temporally overlapped to trials containing rectangle cues as well (an effect that may not have occurred from number cues in Experiment 1 given their lower rate of occurrence compared to letters in the central stream). In a study that employed the same paradigm used here (with only rectangle cues), Santangelo et al. (2011) found that the temporal factor of cue stimulus-onset-asynchrony (SOA) could also cause irrelevant features to be processed. Although such temporal factors were not directly manipulated in this study, future studies should examine the full extent of their influence and possible interaction with elements of the attention set.

Another potential factor that may have influenced the results in Experiment 2 is the relationship of the letter cues to the letters in the central stream. Here the letter cues were selected from the same subset of 17 letters that could occur in the central stream. Although the letter cues were selected to avoid for overlap with the letters in the central stream during that trial, it may be the case that exposure to the same letter on different trials could have been enough to cause facilitation and the subsequent cuing effects observed in Experiment 2. Thus, future studies may also want to investigate such potential interactions by using letter cues selected from a different subset of letters than the central stream.

Overall, we saw evidence of attentional capture for both numbers and letter cues, consistent with the prediction that the attention set determines which object properties can capture attention. Indeed, it is likely that the cuing effect, which is essentially a space based effect, was driven by this sharing of object features between targets and cues in Experiment 1. In this study, establishing the central task as the main focus through higher rate of occurrence (67%) could be equivalent to putting the central task in the attention set. Although participants were only required to respond to the numbers in the central task, each object within the stream had to be processed up to a categorical level in order to successfully perform the task. Thus, the object properties of both numbers and letters were identified in the attention set and subsequently allowed those peripheral cues with similar properties to capture attention. Critically however, the properties of the different objects (numbers and letters) within the attention set appear to affect the attentional capture of peripheral cues along different object and spatial dimensions. Specifically, the attentional capture in Experiment 1 was restricted only to object-based properties, where cuing effects were limited only to number cues, whereas attentional capture in Experiment 2 took on a spatial dimension as well, with cuing effects being observed for the rectangle cues that occurred in the same spatial location as the letter cues.^3^

A holistic picture of the attention set likely involves both top-down and bottom-up signal processing. That is, where the selection and detection of numerical objects within the central stream could invoke top-down control and monitoring possibly via signal amplification, statistical sensitivity to the frequently occurring letters could also generalize beyond their specific object properties to their spatial properties, thereby triggering a more bottom-up orienting response that extended to the spatially-congruent rectangle cues as well.^4^

Our findings here are directly in line with fMRI (functional magnetic resonance imaging) studies indicating that feature-based attending can have a more global effect on stimuli processing, where attending to a particular feature actually increases visual cortical responses to a spatially distant ignored stimulus sharing similar features with the attended stimulus (Saenz et al., 2002). Overall, our findings are also consistent with studies on feature-based attention showing that within the neural pathway, attention to features can occur both independently from, and as early as, spatial processing (contrasting with the more traditional view that selection of non-spatial information subserves spatial processing, see Zhang and Luck, 2009). Other studies have similarly shown how attending to features can also have an early modulatory role on subsequent selectivity (Maunsell and Treue, 2006). Most importantly, such findings are complementary both to the results obtained here as well as the general framework of the attention set. While separate neuroimaging studies have looked at the interaction of both feature and space-based attention as well as top-down-bottom-up processing, combining these multiple dimensions and examining the neural substrates of their interaction may provide vital insights into how attention operates under real world circumstances.

In summary, this study has answered as well as raised important questions that should be examined in future studies designed to investigate and manipulate specific parameters within the attention set. These parameters include the role of different object properties, as well as the ratios and relationships of statistical frequencies of stimuli. For example, although objects within the attention set in this study did not overlap in their categorical features, other studies employing RSVP streams have constructed targets in such a manner that they also overlapped in some way with the distractors (Visser et al., 2004). Typically, such manipulations usually show that detection performance is adversely affected for the target (Visser et al., 2004), however the question remains as to how this configuration may affect the peripheral processing of objects outside the attention set. The question also remains as to whether the attention set operates differently under different types of dual-task paradigms. Whether attention is viewed as an unitary construct or an aggregate of interacting mechanisms, its complex nature likely requires an understanding of how cognitive resources are simultaneously allocated in parallel along multiple dimensions (such as object and spatial). Theories instantiated at the verbal level such as Most et al.’s (2005) may be useful as an initial guiding framework, however, future work may also benefit from the more explicit approaches of computational or neural network modeling that could potentially account for the interaction of such multifaceted mechanisms in a more tractable and precise fashion.

## Conflict of interest statement

The authors declare that the research was conducted in the absence of any commercial or financial relationships that could be construed as a potential conflict of interest.

## Footnotes

^1^Using an inclusion criteria for performance with a mean accuracy rate of 80% or higher and reaction times that were less than 2 s on at least 90% of the trials.

^2^Note that on each trial the number cues were different than the numbers presented in the central RSVP task, in order to avoid potential confounds.

^3^It should be noted that planned *t*-tests between Experiment 1 and 2 for each cell within the experimental design for spatial trials did not reveal any significant differences for RT data. Comparison of the accuracy data revealed lower accuracy scores when invalid number cues were present in Experiment 1 (78%) as compared to when invalid letter cues were present in Experiment 2 (88%), *t*(52) = 8.3, *p* = 0.006. This finding suggests that number cues may have been more distracting than letter cues on spatial trials. Such reduction in accuracy could be due to response mapping interference, where the presence of numbers on a spatial trial may have been more likely to induce a (incorrect) digit response.

^4^This notion involves a two-step explanation. First, the more frequent occurrence of letters in the central task causes more frequent firing for the letter cues in the side as well (feature based association). Once the firing rate for the letter cues increases, the associated or yoked spatial nodes would be more likely to fire on the other 50% of trials where rectangle cues are presented in the same location.
